# Illicit Prescription Opioid Use Among U.S. Firefighters

**DOI:** 10.3390/fire8030112

**Published:** 2025-03-14

**Authors:** Richard R. Suminski, Sara A. Jahnke, Natinee Jitnarin, Christopher Kaipust, Christopher K. Haddock, Walker S. C. Poston

**Affiliations:** 1 Department of Health Behavior and Nutrition Sciences, University of Delaware, Newark, NJ 19716, USA; 2 Center for Fire, Rescue, and EMS Health Research, NDRI-USA, Inc., New York, NY 10001, USA

**Keywords:** fire service, drug use, self-medication, mental health

## Abstract

Firefighters are vulnerable to opioid misuse given the adverse effects their occupation has on mental and physical health. Yet there are limited data on opioid misuse within this population. This study examined the prevalence of illicit prescription opioid use among a nationally representative sample of U.S. firefighters and factors related to opioid misuse. Data were collected through reliable questionnaires from 617 firefighters prior to participating in an intervention designed to mitigate the negative impacts of trauma. The lifetime prevalence of illicit prescription opioid use was 14% compared to 13% in the general U.S. population. The most commonly misused opioids were hydrocodones with trade names Vicodin, Lortab, and Lorcet (72% of those illicitly using opioids). Illicit prescriptions opioid use was not significantly correlated with any demographics examined. However, firefighters who engaged in illicit opioid use exhibited poorer mental health, more alcohol-related problems, and an increased likelihood of misusing other prescription medications. In a regression analysis, alcohol consumption issues, Post-Traumatic Stress Disorder (PTSD), and the illicit use of sedatives and tranquilizers emerged as significant predictors of illicit prescription opioid use. Illicit prescription opioid use by firefighters is a potential problem especially when considered along with other factors such as mental health. Longitudinal studies are needed to further deepen our knowledge about this issue.

## 1. Introduction

Opioids are frequently prescribed to manage acute pain, chronic pain, cancer-related pain, post-surgical pain, vascular pain and for palliative or end-of-life care [1]. These substances work by interacting with opioid receptors in the brain and body, thereby altering the perception and experience of pain. Opioids can be classified as natural, synthetic, or semi-synthetic; common prescription opioids include hydrocodone, oxycodone, codeine, and morphine [2]. While effective in pain relief, opioids can also produce side effects such as euphoria, nausea, sedation, confusion, constipation, tolerance, and dependence.

Illicit prescription opioid use encompasses any use that deviates from a doctor’s instructions, which includes the following: (1) using someone else’s prescription; (2) taking higher doses, using them more frequently, or for a longer duration than prescribed; or (3) consuming them in any manner not directed by a physician [3]. Opioid misuse often stems from their mood-altering effects, physical dependence, or addiction to the drug. The feelings of euphoria associated with opioid use further contribute to the likelihood of misuse [4].

In the United States (U.S.), the prevalence of self-reported lifetime illicit prescription opioids use is around 13% [5,6]. Opioid misuse accounts for the majority of drug overdose fatalities with illicit prescription opioid use constituting approximately a fifth of overdose deaths involving all opioids [7,8]. Research indicates that the use and misuse of opioids vary across different demographics, including age, gender, employment status, race/ethnicity, and income level [9–11]. Additionally, prescription opioids are frequently used illicitly alongside other substances, such as tranquilizers and alcohol [12,13]. Illicit use is also linked to physical pain and mental health disorders, including Post-Traumatic Stress Disorder (PTSD), depression, and anxiety [9,14–17].

Firefighters engage in a variety of dangerous job tasks, such as responding to rescue and fire operations, natural disasters, domestic threats, and providing emergency medical services [18]. Due to the nature of their profession, firefighters face a higher risk of physical injuries and mental health disorders compared to other occupations [19–22]. Research indicates that the prevalence of depression, anxiety, and PTSD among firefighters is greater than that in the general population [23,24]. The extent of these issues is influenced by various factors, including years of service—where longer tenure correlates with increased exposure and risk—the type of injury sustained, particularly head injuries, which are associated with higher severity of depression and PTSD symptoms, and patterns of alcohol use, such as binge drinking [22,24–29].

Research on illicit substance use among firefighters is limited, primarily focusing on alcohol misuse or addressing concerns related to COVID-19 [24,26,27,29,30]. One study did report that a low percentage (1.2%) of Polish firefighters used illicit drugs [31]. However, the study had several methodological limitations and did not provide data on opioid use specifically. To our knowledge, no studies have specifically examined the use of opioids, illicit or otherwise, by U.S. firefighters.

Given the widespread opioid crisis in the U.S. and the numerous risk factors for opioid abuse noted among firefighters, there is a critical need to fill this gap in the literature. Therefore, the current study examined illicit prescription opioid use among a representative sample of U.S. firefighters and to analyze the relationships between opioid misuse and various demographic, behavioral, and physical/mental risk characteristics.

## 2. Materials and Methods

### 2.1. Study Design and Participants

The current investigation utilizes baseline data from the “Stress First Aid Intervention (SFAI)” study funded by the Department of Homeland Security/Federal Emergency Management Agency [32]. The SFAI is a behavioral health intervention designed to mitigate negative outcomes linked to trauma in the fire service. To recruit participants for the SFAI, various strategies were employed to reach fire departments across the U.S., including website advertisements and word-of-mouth referrals. Interested departments underwent screening interviews to assess their motivation levels and available resources. A diverse sample of departments, varying in both region and size, was selected for the study.

The inclusion criteria were as follows: (1) participation was approved by the department’s chief or their designee; (2) the department had no prior experience with the SFAI program; (3) the department had three or more stations; (4) the department agreed to designate a point-of-contact for collaboration with the research team; and (5) the department was willing to allocate a portion of their accrued overtime related to training as a contribution to the grant’s cost-sharing requirement. For departments with four or fewer stations, all firefighters were invited to participate. For departments with more than four stations, four stations were randomly selected to participate in the study.

All firefighters from eight career fire departments and two volunteer fire departments chosen for this study were invited to participate. Those who agreed provided informed, written consent and filled out an online questionnaire that evaluated basic demographic and occupational details, such as years of service and rank, as well as their exposure to traumatic events. Additionally, the questionnaire included inquiries about mental and physical health, alcohol consumption, and the use of illicit substances. Complete data were obtained between 2016 and 2017 on 617 firefighters. The study was conducted according to the guidelines of the Declaration of Helsinki and approved by the Institutional Review Board of NDRI-USA.

### 2.2. Measures

#### 2.2.1. Illicit Drug Use

Questions related to drug use were obtained from the National Survey on Drug Use and Health (NSDUH) [33]. The NSDUH provides nationally representative data annually on current drug, mental, and other health related issues in the U.S. For this study, illicit drug use was defined as ever once using any prescription not prescribed to you or that was taken for the experience or feeling it caused. Participants were shown a list of drugs organized by type (opioids, sedatives, tranquilizers, and stimulants) and asked to select those they used illicitly. They were asked about illicitly using 21 different opioids, 11 different sedatives, 16 different tranquilizers, and 15 different stimulants.

#### 2.2.2. Mental and Physical Health

##### Post-Traumatic Stress Disorder Checklist–Civilian (PCL-C):

The PCL-C modified version previously utilized with firefighters, was used to assess symptoms of post-traumatic stress [34,35]. The PCL-C contains 17 items pertaining to the degree a participant felt bothered by problems with line-of-duty stressful experiences during the past month. Response options and affiliated scores were as follows: not at all = 1; a little bit = 2; moderately = 3; quite a bit = 4; and extremely = 5. The PCL-C demonstrates high internal consistency (α = 0.92), strong two-week test–retest reliability (r = 0.66), and high convergent and discriminant validity when compared to interview-based diagnosis of PTSD symptoms [36].

##### The Life Stress Inventory (LSI):

The LSI is a commonly used tool for assessing total life stress [37]. Participants were presented with a list of 43 stressful events and asked to indicate whether an event happened to them during the past year.

##### Patient Health Questionnaire (PHQ):

The reliable and valid PHQ was utilized to examine physical pain, depression, and anxiety [38]. For physical pain, participants indicated the degree to which they were bothered (not bothered = 0; bothered a little = 1; bothered a lot = 2) during the last four weeks by pain in five body areas-stomach, back, arm/leg/joint, head, chest. Regarding anxiety, participants specified how often over the last four weeks they were bothered (0 = not at all; 1 = several days; 2 = more than half the days) by any of seven anxiety-related symptoms (e.g., nervousness, restlessness, concentration, etc.). Depression was assessed by asking participants to indicate how often during the past two weeks they were bothered by nine depression-related problems. Responses and affiliated scores were as follows: not at all = 0; several days = 1; more than half the days = 2; and nearly every day = 3.

#### 2.2.3. Alcohol Use

Participants indicated how many days during the past month they had at least one alcoholic beverage (12-ounce beer, a 5-ounce glass of wine, or a drink with one shot of liquor) as well as the number of alcoholic beverages they had on the days they drank alcoholic beverages during the past month. In addition, a four-item, YES or NO response, CAGE questionnaire was used to provided information on attributes of problem drinking (e.g., drinking first thing in the morning to steady nerves or get over a hangover) [39]. The possible range of scores is from 0–no alcohol use problems to 4 several alcohol use problems. A total score of two or higher was considered clinically significant and indicative of a need for further evaluation.

### 2.3. Statistical Analysis

Descriptive statistics are presented as mean +/− standard deviation (SD) for continuous variables and percentages for categorical variables. The dependent variable was illicit prescription opioid use coded as 0 = no illicit use and 1 = illicit use. Chi Square analyses for categorical variables and Student’s Independent *t*-tests for continuous variables were used to compare the independent variables between the two levels of the dependent variable. The independent variables assessed with the *t*-tests were all normally distributed (skewness statistic < 1). Binary logistic regression was used to predict illicit prescription opioid use. Predictor variables in the model included the summary scores for anxiety, PTSD, stress, depression, problem drinking (CAGE), and being bothered by pain during the last month along with age and the illicit use of sedatives, tranquilizers, and stimulants coded 0 for no illicit use and 1 for illicit use. Alpha was set a priori at 0.05 and all analyses were performed with the SPSS statistical software package (IBM Corp. Released 2020. IBM SPSS Statistics for Windows, Version 27.0. IBM Corp. Armonk, NY, USA).

## 3. Results

The overall prevalence rate of illicit prescription opioid use was 14% (87/617). Results concerning the breakdown of illicit use according to the type of prescription opioid is provided in Table 1. The most commonly illicitly used prescription opioids were the hydrocodones with trade names Vicodin, Lortab, and Lorcet (72.4%) followed by the oxycodones with trade names Percocet, Percodan, Oxycontin, or Tylox (63.2%), codeien trade name Tylenol #3 (33.3%), and the propoxyphenes with trade names Darvocet and Darvon (23.0%). Less than 7.0% of participants used meperidine trade name Demerol, hydromorphone trade name Dilaudid, morphine, and Methadone.

Overall, participants were 21 to 77 years of age (M = 38.3, SD = 9.1 years) and had one to 44 years of employment in the fire service (M = 13.8, SD = 8.7 years). Participants were mostly White (74.3%), married or cohabitating (89.8%), and educated with 90.9% having some college or a college degree. In addition, 56.1% of the participants were firefighters/paramedics and 67.6% earned $75,000 or more per year. The rates of illicit prescription opioid use according to participant demographics are shown in Table 2. There were no statistically significant differences in illicit use based on any demographic or occupational factors (see Table 2).

Participants illicitly used other prescription drugs including sedatives (2.3%), tranquilizers (7.8%), and stimulants (4.7%) with 19.6% illicitly using two or more drugs. Most (88.7%) consumed at least one alcoholic beverage in the past 30 days and 23.9% scored two or higher on the Cage questionnaire indicating a potential alcohol use problem. The illicit use of other drugs and problem use of alcohol were found to be significantly related to illicit prescription opioid use (Table 3). This was especially evident for sedatives and tranquilizers where 85.7% of illicit sedative users and 60.7% of illicit tranquilizer users also illicitly used prescription opioids.

Participants bothered and not bothered by physical pain illicitly used prescription opioids. However, significantly higher pain summary scores were observed in illicit versus non-illicit prescription opioid users (Table 4). The four mental health indicators examined differed significantly between illicit and non-illicit users of prescription opioids (Table 4). Illicit users were more likely to have symptoms of anxiety, PTSD, life stress, and depression than participants who did not illicitly use prescription opioids.

A binary logistic regression model was significant [F(9519) = 112.6; *p* < 0.001], indicating that our model distinguished between participants not using and using prescription opioids illicitly. The full model explained 19.2 to 34.2% of the variance in illicit prescription opioid use, and correctly classified 86.6% of cases. Four of the nine predictors made a significant contribution to the model (Table 5). Participants who illicitly used sedatives and tranquilizers, had more symptoms of PTSD, and displayed more alcohol use problems were more likely to also illicitly use prescription opioids. It should be noted that although illicit sedative use was a significant predictor, confidence in the estimate is low given the broad 95% confidence interval range.

## 4. Discussion

The national prevalence of illicit opioid use in the U.S. has been on the rise, with opioids now responsible for the majority of drug overdose deaths [40]. The relationships between mental health disorders and opioid misuse have been established in the general population [9,14–17]. In addition, excessive alcohol consumption, occupational stress, and physical pain are considered risk factors for opioid misuse [12,13,15,41]. Although evidence indicates that firefighters are more likely to encounter these risk factors in the line of duty than the general population, there has been limited research specifically focusing on the illicit use of opioids among this group [19–22,24,31]. The results of the current study indicate that illicit prescription opioid use is a potential problem in the fire service and that illicit use is related to mental health, problem drinking, and the illicit use of other substances.

The prevalence of illicit prescription opioid use among the firefighter cohort in the present study was found to be 14%. This figure represents lifetime use and is closely aligned with the 13.6% prevalence reported in the National Drug Use and Health Survey among U.S., non-institutionalized adults and the 13% lifetime nonmedical use observed among high school seniors [5,6]. Previous studies have documented usage of illicit opioid use for various occupational groups, including military personnel, combat veterans, and protective service workers, but none have focused on firefighters [42–46]. One study did report that the use of illicit drugs was only 1.2% among Polish firefighters [31]. However, the researchers did not clarify what illicit drugs were examined and there were methodological weaknesses that reduced the strength of their findings. Considering the lack of evidence regarding illicit prescription opioid use among firefighters, the findings of the present study are significant and highlight the need for further investigation in this area.

Prescription opioids are frequently used in combination with other substances, such as tranquilizers and alcohol [12,13,46–49]. Research by Bohm and Feder [47] indicates that individuals who fit the criteria for opioid abuse are more than twice as likely to misuse tranquilizers. Consistent with these findings, our study found that firefighters who engaged in illicit prescription opioid use were more likely to experience alcohol-related issues and misuse other drugs, particularly tranquilizers. The concurrent use of opioids and other substances heightens the risk of adverse outcomes, including potentially fatal overdoses [48,49]. For instance, consuming opioids in combination with other central nervous system depressants—like benzodiazepines, alcohol, or xylazine—increases the risk of life-threatening overdose [50,51]. Co-use of opioids and alcohol is linked to poorer treatment results for both substances in addition to complicating the management of chronic pain [52]. For firefighters, substances like alcohol may offer temporary relief from the mental and physical challenges of their demanding profession [24]. Yet, substance use has the potential to negatively impact job performance and exacerbate the already high injury rates among firefighters [53]. The most concerning alcohol-related behaviors reported by both career and volunteer firefighters were arriving to work intoxicated, engaging in physical altercations, neglecting responsibilities, and driving after consuming four drinks [53,54]. Additionally, alcohol consumption among firefighters has been closely linked to physical activity levels and hypertension [55]. The consistent patterns observed in both the general population and our findings regarding illicit prescription opioid use in conjunction with other substances among firefighters suggest that interventions aimed at addressing opioid use should also consider the involvement of other substances when evaluating potential treatments or outcomes.

Research indicates a bidirectional relationship between opioid use and mental health disorders like depression, anxiety, and stress [17,56,57]. For instance, among the approximately 39 million adults in the U.S. with mental health disorders, 18.7% use prescription opioids [58]. Furthermore, PTSD is a risk factor for the illicit use of opioids among both the general population and veterans [43,59]. In the current study, firefighters who engaged in illicit prescription opioid use reported significantly higher levels of anxiety, stress, depression, and PTSD compared to those who did not misuse these substances. Notably, PTSD remained a significant predictor of illicit opioid use even after controlling for other factors. Firefighters routinely operate in high-stress environments, confront traumatic incidents, and endure significant physical demands, placing them at heightened risk for both mental and physical health issues [19–22,25]. The interplay between opioid use and these health issues raises concerns, as many firefighters may self-medicate to help with pain relief related to job injuries or to cope with the psychological fallout from traumatic experiences [60,61]. Additionally, the use of illicit substances can heighten psychological vulnerability and increase the likelihood of trauma exposure potentially leading to severe outcomes such as suicidal behaviors [62,63]. Therefore, recognizing the intersection of opioid use and health is essential for creating targeted interventions that support firefighters in managing their physical and mental well-being while dealing with the demands of their profession.

The strengths of this study include the selection of participants from a national sample of fire departments involved in a cluster randomized clinical trial and encompassing a range of regions and sizes. Another notable strength of this research lies in the utilization of reliable and valid questionnaires, representing a methodological advancement over previous studies on during opioid among firefighters [31]. This enhancement increases the accuracy, generalizability, and relevance of the findings. Conversely, there are limitations that must be acknowledged when interpreting the results. The study’s participants were predominantly White, highly educated, and had above-average incomes. While this demographic profile aligns with other studies on firefighters, such homogeneity may have restricted our ability to detect significant demographic variation in illicit prescription opioid use [19,24,64]. This stands in contrast to existing literature, which indicates that opioid use differs across specific demographic groups [9–11]. While obtaining a demographically diverse sample of firefighters in the U.S. could prove challenging with respect to certain demographics such as education and income, purposive sampling could help enhance racial diversity and ensure adequate sub-sample sizes for statistical analysis. Another common limitation in large-scale studies is the potential for bias arising from self-reported data. Self-reported characteristics and behaviors may suffer from recall bias or social desirability bias, potentially leading to both over-reporting and under-reporting, the latter a particular problem when reporting illicit activities like drug use [65,66]. Nevertheless, recent research indicates a high level of agreement between self-reports and biological testing for measuring illicit drug use, suggesting that both methods provide valid insights. However, this accuracy may be limited to more recent use (e.g., past year) if there are problems with self-disclosure [67]. Thus, future research should explore the alignment between biological testing and self-report data on illicit opioid use among firefighters, especially given findings that indicate low sensitivity of surveys in capturing socially undesirable behaviors within this group [68]. Finally, this study emphasized lifetime illicit drug use, allowing for a single instance of use to qualify. While it is often preferable to focus on more recent drug use histories when examining comorbidities related to illicit drug use, our findings demonstrate that broader patterns of use still showcase significant connections between illicit drug use and comorbidities.

## 5. Conclusions

In summary, this study provides evidence that the prevalence of illicit prescription opioid use among firefighters is comparable to that found in the general population, and is linked to mental health issues, alcohol consumption problems, and symptoms of PTSD. Further research focusing on firefighters is essential, particularly to validate self-reported instances of illicit opioid use and to explore causal associations. Additionally, it is timely to consider strategies aimed at reducing illicit prescription opioid use among firefighters, especially given their heightened vulnerability due to the demands of their profession.

## Figures and Tables

**Table 1.** Illicit use of prescription opioids.

Pain Medicines: Generic Name-Brand Name (s)	% YES
Any illicit use (87/617)	14.1
Hydrocodone-Vicodin, Lortab, or Lorcet (63/87)	72.4
Oxycodone-Percocet, Percodan, Oxycontin, or Tylox (55/87)	63.2
Codeine-Tylenol #3 (29/87)	33.3
Propoxyphene-Darvocet or Darvon (20/87)	23.0
Meperidine-Demerol (n = 6) Hydromorphone-Dilaudid (n = 5) Morphine (n = 5) Methadone (n = 1)	<7.0% each drug

**Table 2.** Participant demographic by illicit prescription opioid use.

	No Illicit Use	Illicit Use	*p* Values	Test Statistics

Age in years (M +/− SD)	38.1 (9.0)	40.0 (9.2)	0.07	t = −0.18

Time in Fire Service in years (M +/− SD)	13.8 (8.8)	13.8 (8.1)	0.97	t = 0.04

Race (%)				
White	86.6	13.4	0.31	*X*^2^(1552) = 1.05
Non-White	83.1	16.9		

Marital Status (%)				
Married/cohabitating	85.7	14.3	0.53	*X*^2^(1565) = 0.40
Divorced/widowed/separated/not married	88.1	11.9		

Education (%)				
Some School/HS Grad	82.7	17.3	0.28	*X*^2^(2565) = 2.6
Some College	87.9	12.1		
College/Graduate Degree	83.2	16.8		

Income (%)				
<50 K	88.9	11.1		
50–75 K	85.5	14.5	0.94	*X*^2^(3550) = 0.43
75–100 K	86.0	14.0		
>100 K	85.7	14.3		

Rank(%)				
Firefighter/Paramedic	87.3	12.7		
Driver Operator	90.5	9.5	0.21	*X*^2^(3563) = 4.5
Lieutenant/Captain/Chief	81.1	18.9		
Engineer	86.1	12.2		

**Table 3.** Differences in the misuse of other drugs between participants not illicitly using prescription opioids and those illicitly using prescription opioids.

	No Illicit Use of Opioids	Illicit Use of Opioids	*p* Values	Test Statistics

Sedatives (%)				
No	87.6	12.4	<0.001	*X*^2^(1617) = 60.7
Yes	14.3	85.7		

Tranquilizers (%)				
No	91.0	9.0	<0.001	*X*^2^(1617) = 121.1
Yes	39.3	60.7		

Stimulants (%)				
No	87.9	12.1	<0.001	*X*^2^(1617) = 42.4
Yes	44.8	55.2		

Problem drinking (M +/− SD)	0.7 (1.0)	1.5 (1.4)	<0.001	t = −5.0

# of alcoholic drinks past month (M +/− SD)	36.2 (38.3)	71.5 (79.5)	<0.001	t = −3.9

**Table 4.** Mental and physical health.

Variable (M +/− SD)	No Illicit Use	Illicit Use	*p* Values	Test Statistics
Bothered by pain	2.0 (1.7)	2.5 (1.7)	0.01	t = −2.6
Anxiety	2.4 (3.4)	3.4 (3.8)	0.01	t = −2.6
PTSD	26.6 (9.1)	32.1 (12.2)	<0.001	t = −4.0
Life Stress	6.1 (4.3)	7.7 (4.8)	0.002	t = −3.2
Depression	4.1 (4.2)	6.0 (5.1)	0.002	t = −3.2

Summary scores ranges: Pain 0 to 9 (bothered a lot by physical pain in multiple areas); Anxiety 0 to 14 (bothered by all seven symptoms); PTSD 17 to 78 (more symptoms); Lite stress 0 to 27 (many stress-related events); Depression 0 to 27 (highly bothered by depression-related problems).

**Table 5.** Binary logistic regression results.

	*β*	SE	Wald *X*^2^	OR	95% CI	*p* Value
Pain	0.10	0.10	1.00	1.10	0.91–1.34	0.32
Anxiety	−0.08	0.06	1.75	0.92	0.82–1.04	0.19
PTSD	0.06	0.02	7.92	1.06	1.02–1.10	*0.01*
Stress	0.01	0.04	0.06	1.01	0.94–1.09	0.81
Depression	<0.01	0.05	<0.01	1.00	0.90–1.12	0.97
Alcohol	0.39	0.12	10.43	1.47	1.16–1.86	*0.001*
Sedatives	2.84	1.28	4.97	17.14	1.41–208.46	*0.03*
Tranquilizers	2.40	0.38	40.23	11.05	5.26–23.20	*<0.001*
Stimulants	0.92	0.59	2.43	2.52	0.79–8.02	0.12
Constant	−6.11	0.90	46.14	<0.01		*<0.001*

## Data Availability

The raw data supporting the conclusions of this article will be made available by the authors upon request.
